# Supplementary material for: Metabolite Changes in Indonesian Tempe Production from Raw Soybeans to Over-Fermented Tempe
Source: Metabolites. 2023 Feb 17;13(2):300. doi: 10.3390/metabo13020300 (PMC9958738; doi:10.3390/metabo13020300)
Supplement: Supplementary file 1 [file metabolites-13-00300-s001.zip › metabolites-2110237-supplementary.pdf]

Supplementary Materials

Table S1. List of Detected Metabolites in Indonesian *Tempe* Production from Raw Soybean to Over-Fermented *Tempe.*

No.	Amino Acid	Organic Acid	Sugar	Sugar Alcohol	Others	Unknown
1	Alanine	2-Aminobutyric acid	Arabionose	Glycerol	Urea	Unknown 1
2	Asparagine	Malonic acid	Raffinose	Threitol	Phosphate	Unknown 2
3	Aspartic acid	Sinapinic acid	Melezitose	Meso erythritol	Daidzein (isoflavone)	Unknown 3
4	Cystine	Gluconic acid	b-Lactose	Inositol	Genistein (isoflavone)	Unknown 4
5	Glutamic acid	Nicotinic acid	Maltotriose	2-Aminoethanol	Propyleneglycol	
6	Glutamine	Glyceric acid	Panose	Pyrogallol	Allantoin	
7	Glycine	Fumaric acid	Maltose	Galactinol	n-Propylamine	
8	Histidine	Citramalic acid	Gentiobiose	Mannitol	2-Hydroxypyridine	
9	Homocystine	Xylonic acid	Lyxose	Arabitol	n-Butylamine	
10	Homoserine	Malic acid	Melibiose	Sorbitol	3-Hydroxyisovaleric acid	
11	Isoleucine	2-Aminoadipic acid	Trehalose	Xylitol	Adenosine	
12	Leucine	Pyroglutamic acid	Fructose 6-phosphate_2	Lactitol	Uracil	
13	Lysine	Lactic Acid	Fructose 6-phosphate_3		Thymine	
14	Methionine	Threonic acid	Galactose		Inosine	
15	Phenylalanine	Ferulic acid	Glucose		Putrescine	
16	Proline	Uric acid	Sucrose		O-Phosphoethanolamine	
17	Saccharopine	2-Isopropylmalic acid	Fructose		N-Carbamoyl aspartate	
18	Serine	4-Hydroxyphenylacetic acid	Mannose		Hypoxanthine	
19	Threonine	4-Hydroxybenzoic acid	Ribose		Argininosuccinate	
20	b-Alanine	N-Acetyl aspartic acid	Sorbose		Methionine sulfoxide	
21	Tryptophan	Pipecolic acid	Sugar$_1$		Adenine	
22	Tyrosine	a-Ketoglutaric acid			Glycylglycine	
23	Valine	Glycolic acid			2,6-Diaminopimelate	
24		Shikimic acid			2-Dehydro gluconate	
25		3-Hydroxyanthranilic acid			Pantothenate	
26		2,6-Pyridinedicarboxylic acid			Xanthine	
27		4-Aminobutyric acid			Glucarate	
28		Myristic acid			Tryptamine	
29		Sinapic acid			Uridine	
30		3-Hydroxy-3-methylglutarate			4-Hydroxyphenethyl alcohol	
31		Urocanate				

Table S2. List of Detected Metabolites, Retention Time, and Relative Intensity in Indonesian Tempe Production from Raw Soybean to Over-Fermented Tempe.

No	Metabolite Name	Class	RT[a] (min)	RI[b]	Quant Mass (*m/z*)	MSI Level[c]
1	Alanine	Amino Acid	5.5	1107.33	116	1
2	Asparagine	Amino Acid	10.8	1683.16	116	1
3	Aspartic acid	Amino Acid	9.59	1530.80	232	1
4	Cystine	Amino Acid	14.97	2320.67	204	1
5	Glutamic acid	Amino Acid	10.38	1629.39	246	1
6	Glutamine	Amino Acid	11.56	1785.44	156	1
7	Glycine	Amino Acid	7.68	1316.29	147	1
8	Histidine	Amino Acid	12.63	1940.57	154	1
9	Homocystine	Amino Acid	16.23	2555.69	128	1
10	Homoserine	Amino Acid	8.99	1460.14	218	1
11	Isoleucine	Amino Acid	7.54	1301.51	158	1
12	Leucine	Amino Acid	7.32	1279.44	158	1
13	Lysine	Amino Acid	12.62	1939.27	174	1
14	Methionine	Amino Acid	9.58	1529.94	176	1
15	Phenylalanine	Amino Acid	10.48	1642.07	218	1
16	Proline	Amino Acid	7.58	1305.89	142	1
17	Saccharopine	Amino Acid	15.81	2473.74	318	1
18	Serine	Amino Acid	8.18	1370.12	204	1
19	Threonine	Amino Acid	8.44	1397.90	218	1
20	b-Alanine	Amino Acid	8.78	1436.87	248	1
21	Tryptophan	Amino Acid	14.56	2249.36	202	1
22	Tyrosine	Amino Acid	12.75	1957.77	218	1
23	Valine	Amino Acid	6.75	1223.33	144	1
24	2-Aminobutyric acid	Organic Acid	6.27	1178.41	130	1
25	Malonic acid	Organic Acid	6.59	1207.94	147	1
26	Sinapinic acid	Organic Acid	14.59	2255.49	368	1
27	Gluconic acid	Organic Acid	13.29	2042.08	333	1
28	Nicotinic acid	Organic Acid	7.5	1297.29	180	1
29	Glyceric acid	Organic Acid	7.9	1340.03	147	1
30	Fumaric acid	Organic Acid	7.98	1348.44	245	1
31	Citramalic acid	Organic Acid	9.24	1489.45	160	1
32	Xylonic acid	Organic Acid	11.62	1793.53	292	1
33	Malic acid	Organic Acid	9.31	1497.44	147	1
34	2-Aminoadipic acid	Organic Acid	11.12	1726.30	260	1
35	Pyroglutamic acid	Organic Acid	9.61	1533.76	156	1
36	Lactic Acid	Organic Acid	5.02	1064.49	117	1
37	Threonic acid	Organic Acid	9.98	1578.56	147	1
38	Ferulic acid	Organic Acid	13.69	2104.17	338	1
**39**	Uric acid	Organic Acid	13.82	2126.04	441	1
40	2-Isopropylmalic acid	Organic Acid	10.11	1593.77	275	1
41	4-Hydroxyphenylacetic acid	Organic Acid	10.52	1647.52	179	1
42	4-Hydroxybenzoic acid	Organic Acid	10.43	1635.70	267	1
43	N-Acetyl aspartic acid	Organic Acid	10.66	1665.11	245	1
44	Pipecolic acid	Organic Acid	10.47	1640.19	174	1
45	a-Ketoglutaric acid	Organic Acid	10.02	1583.40	129	1
46	Glycolic acid	Organic Acid	5.17	1078.32	147	1
47	Shikimic acid	Organic Acid	11.81	1820.68	204	1
48	3-Hydroxyanthranilic acid	Organic Acid	12.26	1885.60	354	1

49	2,6-Pyridinedicarboxylic acid	Organic Acid	11.41	1765.85	296	1
50	4-Aminobutyric acid	Organic Acid	9.68	1541.52	174	1
51	Myristic acid	Organic Acid	12.02	1850.39	117	1
52	Sinapic acid	Organic Acid	13.52	2076.97	368	1
53	3-Hydroxy-3-methylglutarate	Organic Acid	10.26	1612.75	147	1
54	Urocanate	Organic Acid	13.11	2012.52	267	1
55	Arabionose	Sugar	10.8	1683.24	103	1
56	Raffinose	Sugar	20.82	3502.04	361	1
57	Melezitose	Sugar	21.08	3600.58	361	1
58	b-Lactose	Sugar	17.35	2780.01	204	1
59	Maltotriose	Sugar	21.6	3682.12	361	1
60	Panose	Sugar	22.28	3771.93	361	1
61	Maltose	Sugar	17.53	2818.35	361	1
62	Gentiobiose	Sugar	17.98	2916.00	204	1
63	Lyxose	Sugar	10.82	1685.03	103	1
64	Melibiose	Sugar	18.11	2945.30	361	1
65	Trehalose	Sugar	17.52	2816.57	361	1
66	Fructose 6-phosphate_2	Sugar	15.16	2355.91	315	1
67	Fructose 6-phosphate_3	Sugar	15.25	2370.94	387	1
68	Galactose	Sugar	12.54	1926.56	319	1
69	Glucose	Sugar	12.58	1931.94	319	1
70	Sucrose	Sugar	16.99	2705.27	362	1
71	Fructose	Sugar	12.46	1914.07	103	1
72	Mannose	Sugar	12.5	1919.90	319	1
73	Ribose	Sugar	10.94	1700.47	103	1
74	Sorbose	Sugar	12.44	1911.69	331	1
75	Sugar[1]	Sugar	12.7	1950.54	319	1
76	Glycerol	Sugar Alcohol	7.34	1282.07	147	1
77	Threitol	Sugar Alcohol	9.46	1515.01	147	1
78	Meso erythritol	Sugar Alcohol	9.53	1523.79	217	1
79	Inositol	Sugar Alcohol	13.86	2131.92	305	1
80	2-Aminoethanol	Sugar Alcohol	7.28	1275.58	174	1
81	Pyrogallol	Sugar Alcohol	9.84	1561.27	292	1
82	Galactinol	Sugar Alcohol	18.69	3076.16	204	1
83	Mannitol	Sugar Alcohol	12.81	1967.36	319	1
84	Arabitol	Sugar Alcohol	11.26	1745.05	117	1
85	Sorbitol	Sugar Alcohol	12.87	1975.65	319	1
86	Xylitol	Sugar Alcohol	11.17	1733.06	217	1
87	Lactitol	Sugar Alcohol	17.65	2844.14	223	1
88	Urea	Others	6.89	1237.64	147	1
89	Phosphate	Others	7.34	1281.75	299	1
90	Daidzein (isoflavone)	Others	18.24	2972.92	398	1
91	Genistein (isoflavone)	Others	18.3	2988.06	471	1
92	Propyleneglycol	Others	4.32	1003.39	117	1
**93**	Allantoin	Others	12.44	1911.91	331	1
94	n-Propylamine	Others	4.6	1027.69	174	1
95	2-Hydroxypyridine	Others	4.73	1039.25	152	1
96	n-Butylamine	Others	5.55	1112.44	174	1
97	3-Hydroxyisovaleric acid	Others	6.66	1214.61	131	1
98	Adenosine	Others	16.81	2668.71	230	1
99	Uracil	Others	7.96	1346.40	241	1

100	Thymine	Others	8.55	1410.28	255	1
101	Inosine	Others	16.49	2605.62	204	1
102	Putrescine	Others	11.34	1755.69	174	1
103	O-Phosphoethanolamine	Others	11.69	1803.31	299	1
104	N-Carbamoyl aspartate	Others	11.75	1812.46	257	1
105	Hypoxanthine	Others	11.8	1819.94	265	1
106	Argininosuccinate	Others	17.12	2732.92	142	1
107	Methionine sulfoxide	Others	12.19	1875.58	128	1
108	Adenine	Others	12.25	1884.40	264	1
109	Glycylglycine	Others	11.91	1835.45	248	1
110	2,6-Diaminopimelate	Others	13.06	2004.99	272	1
111	2-Dehydro gluconate	Others	12.52	1923.68	349	1
112	Pantothenate	Others	13.11	2013.74	291	1
113	Xanthine	Others	13.28	2039.29	353	1
114	Glucarate	Others	13.4	2058.78	333	1
115	Tryptamine	Others	14.47	2233.87	218	1
116	Uridine	Others	15.83	2478.31	217	1
117	4-Hydroxyphenethyl alcohol	Others	10	1580.82	174	1
118	Unknown 1	Others	4.87	1051.49	174	1
119	Unknown 2	Others	11.94	1839.71	273	1
120	Unknown 3	Others	12.39	1903.75	103	1
121	Unknown 4	Others	12.4	1906.21	349	1

There are 121 metabolites detected in all soybean samples from tempe production. a. Retention time in minute(s); b. Retention indices (RI) are calculated using a standard alkane mixture (C9–C40);. c. Mass spectra of metabolite peaks that are compared only with Metabolic Standards Initiative (MSI) the in-house library are considered level 1 identification.

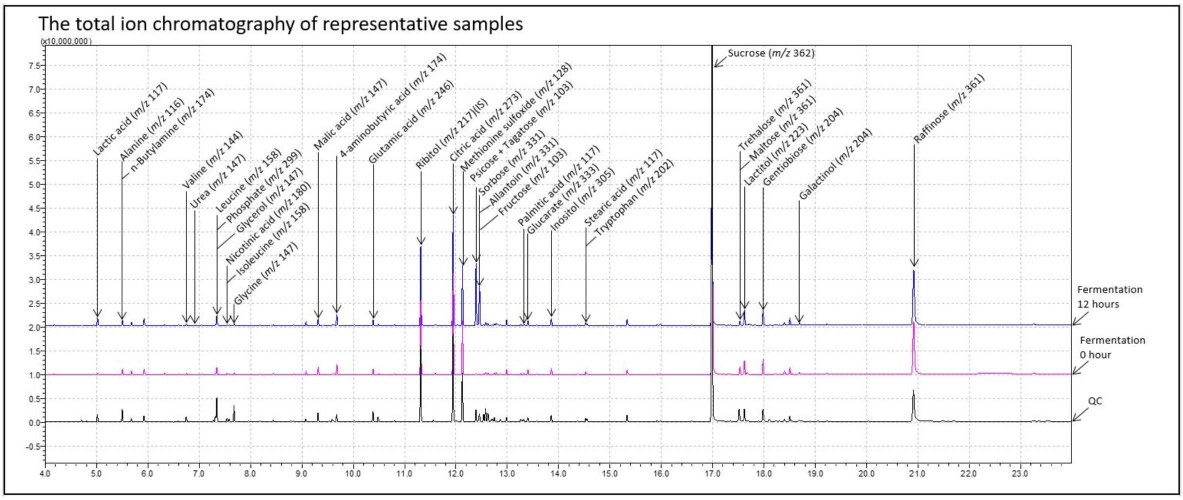

Figure S1. The total ion chromatography of representative samples.

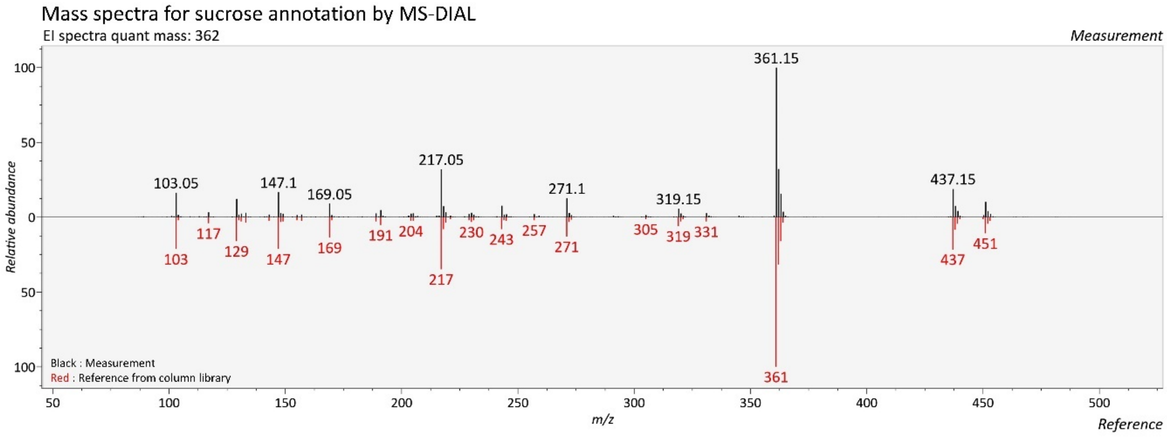

Figure S2. Mass spectra for sucrose annotation by MS-DIAL.

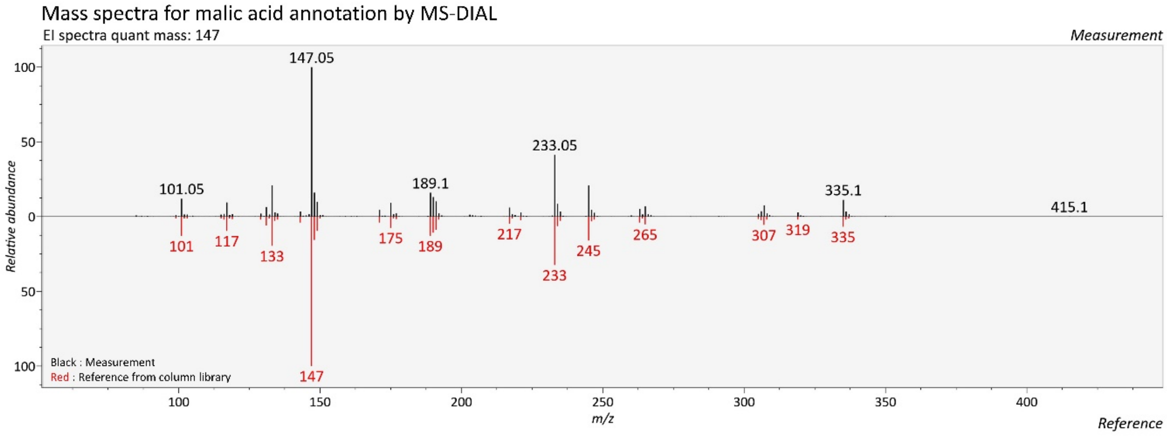

Figure S3. Mass spectra for malic acid annotation by MS-DIAL.

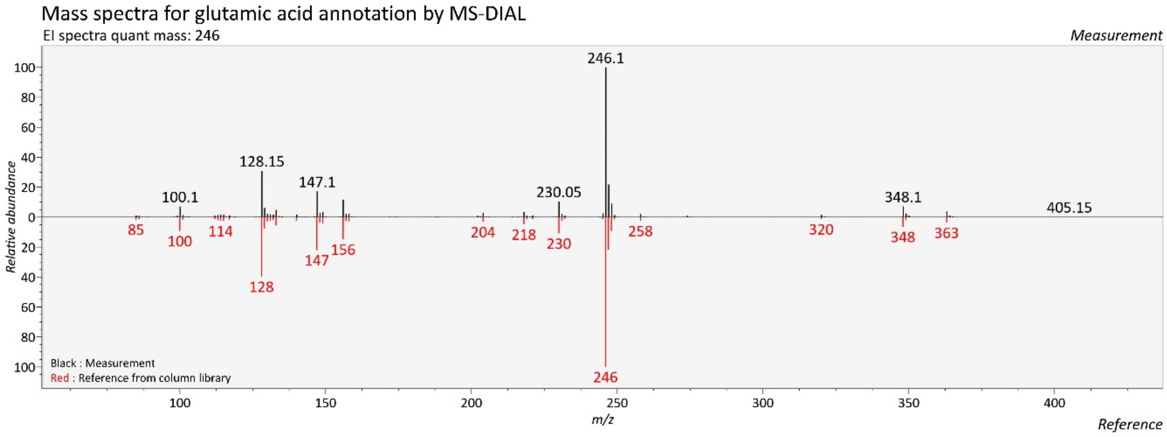

Figure S4. Mass spectra for glutamic acid annotation by MS-DIAL.
